# Dipotassium samarium(III) molybdate(VI) phosphate(V), K_2_Sm(MoO_4_)(PO_4_)

**DOI:** 10.1107/S1600536809041622

**Published:** 2009-10-17

**Authors:** Dan Zhao, Feifei Li, Wendan Cheng, Hao Zhang

**Affiliations:** a Department of Physics and Chemistry, Henan Polytechnic University, Jiaozuo, Henan 454000, People’s Republic of China; b State Key Laboratory of Structural Chemistry, Fujian Institute of Research on the Structure of Matter, Chinese Academy of Sciences, Fuzhou, Fujian, 350002, People’s Republic of China

## Abstract

The title compound, K_2_Sm(MoO_4_)(PO_4_), has been prepared under atmospheric conditions using a high temperature solution growth (HTSG) method. The structure of K_2_Sm(MoO_4_)(PO_4_) is isotypic with other *A*_2_ *M*(MoO_4_)(PO_4_) compounds, where *A* = Na or K, and *M* = trivalent rare earth cation. It can be described as being built up from two-dimensional anionic layers with composition [Sm(MoO_4_)(PO_4_)]^2−^ that are stacked along the *c *axis and are inter­connected by K^+^ cations which are in an eightfold coordination by O atoms. The SmO_8_, MoO_4_ and PO_4_ polyhedra exhibit 2 symmetry.

## Related literature

The structures and properties of other molybdate-phosphates with the general formula *A*_2_ *M*(MoO_4_)(PO_4_), where *A *= Na, K; *M *= Y, Bi, La—Nd, Sm—Lu, have been reported by Ben Amara & Dabbabi (1987 ▶); Komissarova *et al.* (2006 ▶); Ryumin *et al.* (2007 ▶); Zatovsky *et al.* (2006 ▶). For crystallographic background, see: Spek (2009 ▶).

## Experimental

### 

#### Crystal data

K_2_Sm(MoO_4_)(PO_4_)
*M* *_r_* = 483.46
Orthorhombic, 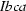

*a* = 12.345 (9) Å
*b* = 7.004 (5) Å
*c* = 19.733 (12) Å
*V* = 1706 (2) Å^3^
*Z* = 8
Mo *K*α radiation
μ = 9.46 mm^−1^
*T* = 298 K
0.15 × 0.10 × 0.05 mm

#### Data collection

Rigaku Saturn 70 diffractometer
Absorption correction: multi-scan (*CrystalClear*; Rigaku, 2000 ▶) *T*_min_ = 0.742, *T*_max_ = 1.000
3660 measured reflections
979 independent reflections
903 reflections with *I* > 2σ(*I*)
*R*_int_ = 0.022

#### Refinement

*R*[*F* ^2^ > 2σ(*F* ^2^)] = 0.014
*wR*(*F* ^2^) = 0.079
*S* = 1.01
979 reflections
61 parameters
Δρ_max_ = 0.85 e Å^−3^
Δρ_min_ = −0.99 e Å^−3^

### 

Data collection: *CrystalClear* (Rigaku, 2000 ▶); cell refinement: *CrystalClear*; data reduction: *CrystalClear*; program(s) used to solve structure: *SHELXS97* (Sheldrick, 2008 ▶); program(s) used to refine structure: *SHELXL97* (Sheldrick, 2008 ▶); molecular graphics: *SHELXTL* (Sheldrick, 2008 ▶) and *DIAMOND* (Brandenburg & Putz, 2005 ▶); software used to prepare material for publication: *SHELXTL*.

## Supplementary Material

Crystal structure: contains datablocks I, global. DOI: 10.1107/S1600536809041622/wm2263sup1.cif

Structure factors: contains datablocks I. DOI: 10.1107/S1600536809041622/wm2263Isup2.hkl

Additional supplementary materials:  crystallographic information; 3D view; checkCIF report

## Figures and Tables

**Table 1:** Selected bond lengths (Å)

K1—O4^i^	2.685 (3)
K1—O2	2.695 (3)
K1—O2^ii^	2.768 (3)
K1—O3^ii^	2.991 (4)
K1—O4	3.012 (4)
K1—O1^iii^	3.025 (3)
K1—O4^iv^	3.184 (4)
K1—O3^v^	3.191 (4)
Sm1—O1^ii^	2.339 (3)
Sm1—O3	2.399 (3)
Sm1—O2	2.451 (3)
Sm1—O1^vi^	2.481 (3)
Mo1—O4	1.743 (3)
Mo1—O3	1.784 (3)
P1—O2	1.533 (3)
P1—O1	1.549 (3)

Symmetry codes: (i) 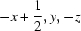; (ii) 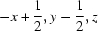; (iii) 
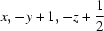
; (iv) 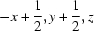; (v) 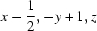; (vi) 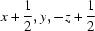.

**Fig. 1.**
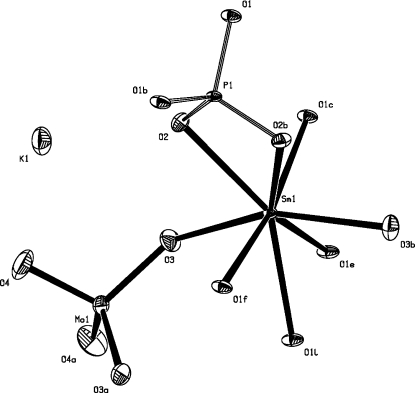
Part of the structure of the title compound, showing the labelling of the atoms. Displacement ellipsoids are drawn at the 50% probability level. [Symmetry codes: (a) - X, 1/2 - Y, Z (b) X, - Y, 1/2 – Z (c) - X, 1/2 + Y, 1/2 – Z (e) X, 1/2 + Y, - Z (f) - X, Y, 1/2 + Z (l) 1/2 + X, Y, 1/2 – z.]

## supplementary crystallographic information

### Comment

In recent years, several complex molybdate-phosphates with the general formula *A*_2_*M*(MoO_4_)(PO_4_) (*A* = Na, K; *M*= Y, Bi, La—Nd, Sm—Lu) have been reported (Ben Amara & Dabbabi, 1987); Komissarova *et al.*, 2006; Ryumin *et al.*, 2007; Zatovsky *et al.*, 2006). Herein, we present synthesis and crystal structure of the title compound, K_2_Sm(MoO_4_)(PO_4_).

X-ray analysis revealed that the compound K_2_Sm(MoO_4_)(PO_4_) crystallizes in the orthorhombic system with space group *Ibca* (Fig. 1). The asymmetric unit contains one K, one Sm, one Mo, one P and four O atoms. Each of the Mo and P atoms is tetrahedrally coordinated by four O atoms, forming nearly ideal MoO_4_ and PO_4_ tetrahedra with two types of Mo–O (1.743 (3) Å, 1.784 (3) Å) and P–O distances (1.533 (3) Å, 1.549 (3) Å), respectively. The Sm atom is surrounded by eight oxygen atoms in a distorted dodecahedral environment with the Sm–O bond distances in the range of 2.339 (3)–2.481 (3) Å. MoO_4_, PO_4_ and SmO_8_ polyhedra are interconnected *via* corner- or edge-sharing O atoms forming a two-dimensional anionic layer of overall composition [Sm(MoO_4_)(PO_4_)]^2-^ extending parallel to the *ab*-plane, as shown in Fig. 2. Furthermore, the anionic framework delimits large cages in which the K^+^ cations (coordination number 8) reside to ensure the cohesion of the structure and the neutrality of the compound, resulting in a three-dimensional framework structure.

### Experimental

Single crystals of K_2_Sm(MoO_4_)(PO_4_) were obtained by the high temperature solution growth (HTSG) reaction of K_2_CO_3_ (2.253 g, 16.3 mmol), Sm_2_O_3_ (0.569g, 1.63 mmol), MoO_3_ (0.939 g, 6.52 mmol) and NH_4_H_2_PO_4_ (3.000 g, 26.1 mmol), with the molar ratio of 10:1:2:8 (K:Sm:Mo:P). The reaction mixture was thoroughly ground in an agate mortar and pressed into a pellet to ensure the best homogeneity and reactivity. The pellet was put into a platinum crucible and calcined at 673 K or 10 h to decompose the salts. Then the crucible was transferred to another furnace and heated at 1223 K for 48 h. Then the sample was cooled from 1173 K at a rate of 4 Kh^-1^. After boiling for 24 h in water, a few prism-shaped colorless crystals were obtained in very low yield (<5%).

### Crystal data

**Table:**

K_2_Sm(MoO_4_)(PO_4_)	*F*(000) = 1768
*M**_r_* = 483.46	*D*_x_ = 3.764 Mg m^−^^3^
Orthorhombic, *I**b**c**a*	Mo *K*α radiation, λ = 0.71073 Å
Hall symbol: -I 2b 2c	Cell parameters from 369 reflections
*a* = 12.345 (9) Å	θ = 3.1–27.4°
*b* = 7.004 (5) Å	µ = 9.46 mm^−^^1^
*c* = 19.733 (12) Å	*T* = 298 K
*V* = 1706 (2) Å^3^	Prism, colourless
*Z* = 8	0.15 × 0.10 × 0.05 mm

### Data collection

**Table:**

Rigaku Saturn 70 diffractometer	979 independent reflections
Radiation source: fine-focus sealed tube	903 reflections with *I* > 2σ(*I*)
graphite	*R*_int_ = 0.022
Detector resolution: 14.6306 pixels mm^-1^	θ_max_ = 27.5°, θ_min_ = 2.1°
ω scans	*h* = −15→15
Absorption correction: multi-scan (*CrystalClear*; Rigaku, 2000)	*k* = −9→8
*T*_min_ = 0.742, *T*_max_ = 1.000	*l* = −25→25
3660 measured reflections	

### Refinement

**Table:**

Refinement on *F*^2^	0 restraints
Least-squares matrix: full	Primary atom site location: structure-invariant direct methods
*R*[*F*^2^ > 2σ(*F*^2^)] = 0.014	Secondary atom site location: difference Fourier map
*wR*(*F*^2^) = 0.079	*w* = 1/[σ^2^(*F*_o_^2^) + (0.063*P*)^2^] where *P* = (*F*_o_^2^ + 2*F*_c_^2^)/3
*S* = 1.01	(Δ/σ)_max_ < 0.001
979 reflections	Δρ_max_ = 0.85 e Å^−^^3^
61 parameters	Δρ_min_ = −0.99 e Å^−^^3^

### Special details

**Table:**

Geometry. All e.s.d.'s (except the e.s.d. in the dihedral angle between two l.s. planes) are estimated using the full covariance matrix. The cell e.s.d.'s are taken into account individually in the estimation of e.s.d.'s in distances, angles and torsion angles; correlations between e.s.d.'s in cell parameters are only used when they are defined by crystal symmetry. An approximate (isotropic) treatment of cell e.s.d.'s is used for estimating e.s.d.'s involving l.s. planes.
Refinement. Single crystal of K_2_Sm(MoO_4_)(PO_4_)with dimensions of 0.15 mm×0.10 mm×0.05 mm was selected for single-crystal X-ray diffraction determination. The diffraction data of K_2_Sm(MoO_4_)(PO_4_) were collected on a Rigaku Saturn70 CCD diffractometer with graphite-monochromated Mo—Ka radiation (λ = 0.71073 Å). Intensity data were collected by the narrow frame method at 298 K. The data were corrected for Lorentz factor, polarization, air absorption and absorption due to variations in the path length through the detector faceplate. Absorption corrections based on Multi-scan technique were also applied (*CrystalClear*. ver. 1.3.5.,Rigaku Corp., Woodlands, TX, 1999). The single-crystal refinement was performed with the program *SHELX97*. The spacegroup was determined to be *Ibca* based on systematic absences as well as E-value statistics. The structure was solved by the direct methods and the molybdenum and phosphorus atoms were revealed. Subsequent difference-Fourier syntheses by full-matrix least-squares fitting on *F^2^* allowed localization of potassium and all oxygen atoms. The final structure refinement performed by least-square methods with atomic coordinates and anisotropic thermal parameters resulted in satisfactory residuals. In addition, the final refined solutions obtained for were checked with the ADDSYM algorithm in the program *PLATON* (Spek, 2009) and no higher symmetries were found.Refinement of *F*^2^ against ALL reflections. The weighted *R*-factor *wR* and goodness of fit *S* are based on *F*^2^, conventional *R*-factors *R* are based on *F*, with *F* set to zero for negative *F*^2^. The threshold expression of *F*^2^ > σ(*F*^2^) is used only for calculating *R*-factors(gt) *etc*. and is not relevant to the choice of reflections for refinement. *R*-factors based on *F*^2^ are statistically about twice as large as those based on *F*, and *R*- factors based on ALL data will be even larger.

### Fractional atomic coordinates and isotropic or equivalent isotropic displacement parameters (Å^2^)

**Table:**

	*x*	*y*	*z*	*U*_iso_*/*U*_eq_	
K1	0.17099 (8)	0.28431 (16)	0.09418 (5)	0.0233 (3)	
Sm1	0.42384 (2)	0.5000	0.2500	0.00627 (15)	
Mo1	0.5000	0.2500	0.08208 (2)	0.01213 (17)	
P1	0.17826 (12)	0.5000	0.2500	0.0066 (4)	
O1	0.0995 (3)	0.6698 (4)	0.26009 (13)	0.0111 (6)	
O2	0.2522 (3)	0.5240 (3)	0.18818 (14)	0.0113 (6)	
O3	0.4690 (3)	0.4533 (5)	0.13294 (14)	0.0177 (6)	
O4	0.3879 (3)	0.1914 (5)	0.03256 (15)	0.0292 (8)	

### Atomic displacement parameters (Å^2^)

**Table:**

	*U*^11^	*U*^22^	*U*^33^	*U*^12^	*U*^13^	*U*^23^
K1	0.0327 (6)	0.0226 (5)	0.0147 (4)	−0.0016 (4)	−0.0042 (4)	−0.0018 (4)
Sm1	0.0049 (2)	0.0050 (2)	0.0090 (2)	0.000	0.000	0.00005 (8)
Mo1	0.0142 (3)	0.0153 (3)	0.0069 (3)	0.00155 (19)	0.000	0.000
P1	0.0041 (8)	0.0042 (9)	0.0115 (8)	0.000	0.000	0.0001 (4)
O1	0.0065 (13)	0.0058 (15)	0.0212 (13)	0.0000 (12)	−0.0006 (11)	−0.0018 (11)
O2	0.0085 (14)	0.0139 (14)	0.0116 (13)	−0.0026 (11)	−0.0010 (12)	0.0021 (10)
O3	0.0228 (18)	0.0192 (15)	0.0112 (13)	0.0032 (14)	0.0029 (12)	−0.0013 (12)
O4	0.032 (2)	0.033 (2)	0.0224 (15)	−0.0012 (16)	−0.0163 (15)	0.0016 (14)

### Geometric parameters (Å, °)

**Table:**

K1—O4^i^	2.685 (3)	Mo1—O4^ix^	1.743 (3)
K1—O2	2.695 (3)	Mo1—O4	1.743 (3)
K1—O2^ii^	2.768 (3)	Mo1—O3	1.784 (3)
K1—O3^ii^	2.991 (4)	Mo1—O3^ix^	1.784 (3)
K1—O4	3.012 (4)	P1—O2	1.533 (3)
K1—O1^iii^	3.025 (3)	P1—O2^iii^	1.533 (3)
K1—O4^iv^	3.184 (4)	P1—O1^iii^	1.549 (3)
K1—O3^v^	3.191 (4)	P1—O1	1.549 (3)
Sm1—O1^ii^	2.339 (3)	O1—Sm1^vi^	2.339 (3)
Sm1—O1^vi^	2.339 (3)	O1—Sm1^x^	2.481 (3)
Sm1—O3	2.399 (3)	O1—K1^iii^	3.025 (3)
Sm1—O3^iii^	2.399 (3)	O2—K1^iv^	2.768 (3)
Sm1—O2	2.451 (3)	O3—K1^iv^	2.991 (4)
Sm1—O2^iii^	2.451 (3)	O3—K1^viii^	3.191 (4)
Sm1—O1^vii^	2.481 (3)	O4—K1^i^	2.685 (3)
Sm1—O1^viii^	2.481 (3)	O4—K1^ii^	3.184 (4)
			
O4^i^—K1—O2	153.25 (10)	O3^iii^—Sm1—O1^vii^	78.98 (10)
O4^i^—K1—O2^ii^	123.87 (11)	O2—Sm1—O1^vii^	133.07 (9)
O2—K1—O2^ii^	79.73 (9)	O2^iii^—Sm1—O1^vii^	145.94 (9)
O4^i^—K1—O3^ii^	83.90 (10)	O1^ii^—Sm1—O1^viii^	68.11 (13)
O2—K1—O3^ii^	121.45 (10)	O1^vi^—Sm1—O1^viii^	126.05 (8)
O2^ii^—K1—O3^ii^	61.05 (10)	O3—Sm1—O1^viii^	78.98 (10)
O4^i^—K1—O4	79.19 (11)	O3^iii^—Sm1—O1^viii^	77.59 (10)
O2—K1—O4	94.69 (10)	O2—Sm1—O1^viii^	145.94 (9)
O2^ii^—K1—O4	79.85 (10)	O2^iii^—Sm1—O1^viii^	133.07 (9)
O3^ii^—K1—O4	116.70 (11)	O1^vii^—Sm1—O1^viii^	58.14 (15)
O4^i^—K1—O1^iii^	146.34 (11)	O4^ix^—Mo1—O4	111.8 (2)
O2—K1—O1^iii^	52.27 (9)	O4^ix^—Mo1—O3	107.33 (15)
O2^ii^—K1—O1^iii^	62.16 (9)	O4—Mo1—O3	109.46 (16)
O3^ii^—K1—O1^iii^	70.78 (8)	O4^ix^—Mo1—O3^ix^	109.46 (16)
O4—K1—O1^iii^	131.75 (9)	O4—Mo1—O3^ix^	107.33 (15)
O4^i^—K1—O4^iv^	78.43 (8)	O3—Mo1—O3^ix^	111.5 (2)
O2—K1—O4^iv^	77.88 (9)	O2—P1—O2^iii^	106.9 (3)
O2^ii^—K1—O4^iv^	157.51 (9)	O2—P1—O1^iii^	110.83 (15)
O3^ii^—K1—O4^iv^	131.29 (10)	O2^iii^—P1—O1^iii^	113.07 (14)
O4—K1—O4^iv^	104.03 (10)	O2—P1—O1	113.08 (14)
O1^iii^—K1—O4^iv^	101.61 (9)	O2^iii^—P1—O1	110.83 (15)
O4^i^—K1—O3^v^	98.70 (10)	O1^iii^—P1—O1	102.3 (3)
O2—K1—O3^v^	76.53 (10)	P1—O1—Sm1^vi^	145.66 (18)
O2^ii^—K1—O3^v^	119.10 (9)	P1—O1—Sm1^x^	99.81 (16)
O3^ii^—K1—O3^v^	86.19 (11)	Sm1^vi^—O1—Sm1^x^	111.05 (13)
O4—K1—O3^v^	156.35 (10)	P1—O1—K1^iii^	91.19 (13)
O1^iii^—K1—O3^v^	58.87 (8)	Sm1^vi^—O1—K1^iii^	90.67 (9)
O4^iv^—K1—O3^v^	52.93 (8)	Sm1^x^—O1—K1^iii^	112.46 (9)
O1^ii^—Sm1—O1^vi^	165.83 (15)	P1—O2—Sm1	96.39 (15)
O1^ii^—Sm1—O3	88.61 (10)	P1—O2—K1	104.95 (15)
O1^vi^—Sm1—O3	94.68 (10)	Sm1—O2—K1	128.43 (12)
O1^ii^—Sm1—O3^iii^	94.68 (10)	P1—O2—K1^iv^	144.03 (15)
O1^vi^—Sm1—O3^iii^	88.61 (10)	Sm1—O2—K1^iv^	94.74 (11)
O3—Sm1—O3^iii^	153.13 (16)	K1—O2—K1^iv^	94.39 (10)
O1^ii^—Sm1—O2	90.20 (9)	Mo1—O3—Sm1	134.48 (17)
O1^vi^—Sm1—O2	77.48 (9)	Mo1—O3—K1^iv^	126.79 (14)
O3—Sm1—O2	74.39 (11)	Sm1—O3—K1^iv^	90.36 (10)
O3^iii^—Sm1—O2	132.14 (11)	Mo1—O3—K1^viii^	99.04 (13)
O1^ii^—Sm1—O2^iii^	77.48 (9)	Sm1—O3—K1^viii^	109.51 (11)
O1^vi^—Sm1—O2^iii^	90.20 (9)	K1^iv^—O3—K1^viii^	86.78 (10)
O3—Sm1—O2^iii^	132.14 (11)	Mo1—O4—K1^i^	132.86 (18)
O3^iii^—Sm1—O2^iii^	74.39 (11)	Mo1—O4—K1	115.37 (15)
O2—Sm1—O2^iii^	60.33 (15)	K1^i^—O4—K1	94.75 (10)
O1^ii^—Sm1—O1^vii^	126.05 (8)	Mo1—O4—K1^ii^	100.26 (13)
O1^vi^—Sm1—O1^vii^	68.11 (13)	K1^i^—O4—K1^ii^	120.73 (12)
O3—Sm1—O1^vii^	77.59 (10)	K1—O4—K1^ii^	80.58 (9)

Symmetry codes: (i) −*x*+1/2, *y*, −*z*; (ii) −*x*+1/2, *y*−1/2, *z*; (iii) *x*, −*y*+1, −*z*+1/2; (iv) −*x*+1/2, *y*+1/2, *z*; (v) *x*−1/2, −*y*+1, *z*; (vi) −*x*+1/2, −*y*+3/2, −*z*+1/2; (vii) *x*+1/2, *y*, −*z*+1/2; (viii) *x*+1/2, −*y*+1, *z*; (ix) −*x*+1, −*y*+1/2, *z*; (x) *x*−1/2, *y*, −*z*+1/2.

## References

[bb1] Ben Amara, M. & Dabbabi, M. (1987). *Acta Cryst.* C**43**, 616–618.

[bb2] Brandenburg, K. & Putz, H. (2005). *DIAMOND* Crystal Impact GbR, Bonn, Germany.

[bb3] Komissarova, L. N., Ryumin, M. A., Bobylev, A. P., Zhizhin, M. G. & Danilov, V. P. (2006). *Russ. J. Inorg. Chem.* **51**, 397–403.

[bb4] Rigaku (2000). *CrystalClear* Rigaku Corporation, Tokyo, Japan.

[bb5] Ryumin, M. A., Komissarova, L. N., Rusakov, D. A., Bobylev, A. P. & Zhizhin, M. G. (2007). *Russ. J. Inorg. Chem.* **52**, 717–724.

[bb6] Sheldrick, G. M. (2008). *Acta Cryst.* A**64**, 112–122. 10.1107/S0108767307043930 18156677

[bb7] Spek, A. L. (2009). *Acta Cryst.* D**65**, 148–155. 10.1107/S090744490804362X PMC2631630 19171970

[bb8] Zatovsky, I. V., Terebilenko, K. V., Slobodyanik, N. S., Baumer, V. N. & Shishkin, O. V. (2006). *J. Solid State Chem.* **179**, 3550–3555.

